# Cost-effectiveness of vaccinating children at school – A commentary

**DOI:** 10.12968/jfch.2025.2.6.256

**Published:** 2025-06-02

**Authors:** Omobolanle Olagunju, Trevor Bayley, Valerio Benedetto, Luís Filipe

**Affiliations:** 1 Health Innovation North West Coast, United Kingdom; 2 National Institute for Health and Care Research Applied Research Collaboration North West Coast, United Kingdom; 3 University of Central Lancashire and National Institute for Health and Care Research Applied Research Collaboration North West Coast, United Kingdom; 4 Division of Health Research, Faculty of Health and Medicine, Lancaster University, United Kingdom

**Keywords:** Influenza, School based vaccination, Cost-effectiveness, Critical Appraisal

## Abstract

Barriers to vaccination can significantly impact the uptake of influenza vaccines among school-aged children. Offering influenza vaccination within schools could reduce these barriers and promote higher vaccination rates.

This commentary critically evaluates an economic evaluation comparing the cost-effectiveness of school-located influenza vaccination (SLIV) programs for elementary and secondary school students against the standard of care, i.e. vaccination in primary care settings.

The economic evaluation suggested that SLIV could be cost-effective, particularly when considering indirect cost savings and spillover effects. However, the commentary highlights an insufficient explanation of the underlying mechanisms of spillover effects in improving cost-effectiveness, and a lack of discussion of similar programs aimed at boosting vaccination rates.

Future research should investigate population preferences, examining why parents and children opt for SLIV or end up taking practice-based vaccination as a spillover effect of the SLIV program. The role played by heterogeneity, exploring factors such as gender, ethnicity, income, and other socioeconomic characteristics should also be investigated to better understand the program’s impact on different population groups.

## Introduction

### Prevalence and impact for the individuals/caregivers

During the 2023-24 flu season, the Centers for Disease Control and Prevention reported that only 57% of children aged 5-12 years and 46.9% of children aged 13-17 year received flu vaccination in the USA, compared to 64.5% and 53.3%, in 2019-2020.^1^

Influenza can cause serious morbidity and mortality, loss of productivity and financial burden. Seasonal influenza spreads easily in schools and in other crowded areas. Influenza infection can result not only in high levels of school absenteeism but also productivity losses of their carers^2^, as symptoms can last up to 2 weeks or longer.^3^

To prevent or reduce the impact of influenza, the World Health Organization recommends that school children and their carers are vaccinated against the virus.^4^

### Economic impact for individuals/healthcare system/wider economy

The overall burden of influenza for the 2023-2024 flu season was an estimated 40 million flu-related illnesses, 18 million flu-related medical visits, 470,000 flu-related hospitalizations, and 28,000 flu-related deaths.^5^

The mean medical cost per ED visit was $512, with annual Emergency Departments cost burden estimated at $62 to $279 million.^6^

### Justification and aim of economic evaluation

One of the barriers to influenza vaccination is the need for the parents and children spend time and money going to a primary care practice. This burden could be reduced by providing influenza vaccination within the school, through the school located influenza vaccination (SLIV) program. Yoo et al. (2019)^7^ conducted the economic evaluation to understand the cost-effectiveness of the SLIV program and how it varies according to age groups (elementary vs secondary aged children).

### Aim of commentary

This commentary aims to critically appraise the methods, and the results reported in the economic evaluation by Yoo et al. 2019 and expand upon the findings in the context of child vaccination programs.

## Methods

The economic evaluation conducted by Yoo et al. (2019) assessed the cost-effectiveness of two SLIV programs in upstate New York during the 2015–2016 season, one in elementary schools and the other in secondary schools.

For elementary schools, a stepped wedge design was employed. In the 2014–2015 school year, 24 suburban and 18 urban schools were divided into control (no SLIV) and treatment (with SLIV) groups (12 suburban and 9 urban each), with samples of 10,185 and 11,511 students, respectively. By 2015–2016, all schools were assigned to the SLIV program. In secondary schools, a cluster randomized trial was used, where 16 suburban and 4 urban schools were assigned to either the control or treatment groups (8 suburban and 2 urban each) in the 2015–2016 school year, with samples of 8,850 and 9,488 students, respectively. School pairings within districts were adjusted based on the percentage of students eligible for free/reduced cost school lunch.

In both elementary and secondary schools, parents were informed about the SLIV program via email (for suburban schools) or backpack fliers (for both suburban and urban schools). Student names and birthdates from school directories were matched with the New York Immunization Information System to gather influenza vaccination data.

Yoo et al. (2019) used decision tree models to perform cost-effectiveness analyses from a societal perspective, considering a one-year time horizon. Most cost and all effectiveness parameters were based on primary data collected during the study. Incremental cost-effectiveness ratios (ICERs) were presented as the cost per additional vaccinated student.

To address uncertainties in the model parameters, the authors conducted a one-way sensitivity analysis and a probabilistic sensitivity analysis using Monte Carlo simulations, assigning distributions to the parameters. The study evaluated two effectiveness measures: (1) the difference in the proportion of students vaccinated “Anywhere” (schools and primary care centres); and (2) the difference in the proportion of students vaccinated in SLIV clinics.

The study estimated three cost components for the SLIV program: (A) school costs, including non-labour material expenses (e.g., supplies, distribution of information to parents), labour costs for school staff (e.g., attending preparatory meetings, escorting students), and the cost of the web-consent system; (B) project coordination costs, covering expenses related to coordinating activities; and (C) vendor costs, which encompassed the vendor’s costs for administering vaccinations (labour for administering SLIV vaccinations and billing insurers) as well as material costs (e.g., vaccine purchase, refrigeration, and medical supplies). Additionally, averted costs (D) were calculated, representing the savings to parents who would otherwise accompany their child to a primary care visit for an influenza vaccination. All costs were reported in 2015 US dollars.

## Results

When excluding averted costs and spillovers (i.e., where SLIV serves as a “reminder” that increases vaccination rates in primary care practices) from the analysis, the ICER estimates for the SLIV program were comparable between elementary and secondary schools ($85.71 vs. $86.51 per additional child vaccinated). When spillover effects to primary care practices were considered, SLIV programs in secondary schools were more cost effective than those in elementary schools ($53.40 vs. $80.53 per additional child vaccinated?). Accounting for averted costs decreased the ICERs for the SLIV program in elementary and secondary schools significantly ($48.90 vs. $49.70 without spillovers, and $46.89 vs. $36.57 with spillovers). In this scenario, the mean ICER for all schools and the ICER for secondary schools was lower than the mean cost of vaccination at primary care practices. The probabilistic sensitivity analysis yielded very similar results.

Previous studies by the same authors from 2009-2010 and 2010-2011,^8,9^ estimated ICERs of around $65 per additional child vaccinated in elementary schools through SLIV, excluding spillover effects. In contrast, the 2015-2016 program for elementary schools showed a higher ICER of $85.71. This increase in the ICER is attributed to the introduction of the web consent system, which added $0.57 per eligible child and $12.97 per additionally vaccinated child, along with increased project coordination expenses amounting to $1.70 per eligible child and $38.81 per additionally vaccinated child. However, when accounting for potential spillover effects differences in ICERs reduced: the 2009-2010 and 2010-2011 programs showed ICERs of $72.56 and $63.35, respectively, while the 2015-2016 program presented a slightly higher ICER of $80.53.

A one-way sensitivity analysis revealed that the ICER estimates were most sensitive to effectiveness parameters, particularly the primary effectiveness measure (vaccination rate among children in SLIV schools), but remained robust against cost parameters.

A break-even analysis indicated that, for SLIV and primary care practice-based vaccinations to achieve the same efficiency, the percentage of vaccinated students in all schools would need to increase from 4.4% to 15.1% when averted parental costs are not considered. If these costs were considered, the required percentage would drop to 11.6%.

## Commentary

### Critical appraisal

We evaluated the study by Yoo et al. (2019) using a checklist comprising selected questions from three widely recognized quality appraisal tools in economic literature.^10–12^ The study effectively identified the decision problem, perspective, model structure, and cost components of the economic evaluation, and it provided a reasonable interpretation of the results (Table 1). Only minor concerns were noted regarding the discussion of certain results.

The authors assert that SLIV programs may be more cost-effective than primary care practice-based vaccinations when both averted costs and spillover effects are considered ($36 vs. $45 per vaccination) (cite). However, they do not elaborate on the mechanisms for this conclusion. Spillover effects—where SLIV serves as a “reminder” that increases vaccination rates in primary care practices—imply that the average costs for primary care vaccinations would need to decrease significantly to offset the higher costs associated with SLIV. A detailed discussion of these cost dynamics and the cost structure of primary care practices would be necessary to help interpreting these results.

Additionally, the authors rely on the average cost of vaccination derived from a stratified random sample of paediatric practices in New York State. In their prior work,^8,13^ they reported substantial variation in vaccination costs, with the mean of costs approximating their 75th percentile. A discussion on the generalizability of this average cost to the specific regions studied would provide valuable context.

Finally, since spillover effects are incorporated into the analysis, the study assumes that SLIV are complementary to practice-based vaccinations. But in that case, the discussion should focus on comparing the cost-effectiveness of SLIV to alternative (substitute) strategies aimed at increasing overall vaccination rates among children (and not to primary care-based vaccinations).

### Implications for policy/practice

The study by Yoo et al. (2019) offers a comprehensive estimate of the cost per additional child vaccinated through the SLIV program. While SLIV is generally more expensive than traditional vaccination methods, it is an effective strategy to increase overall vaccination rates. Policymakers must weigh these higher costs against the benefits of vaccinating more children, such as reduced illness,^14^ lower absenteeism among children^15–17^ and parents,^17–19^ decreased healthcare expenses,^20,21^ and reduced virus transmission within households and communities. ^22,23^

The study also emphasizes the role of “reminders” in SLIV campaigns, which encourage parents or children to seek vaccinations at primary care facilities. This raises a critical question: how effective might other influenza vaccination promotion strategies, such as targeted advertising or alternative reminder systems, be in achieving similar results?^24^ A comparative evaluation of the cost-effectiveness of these alternatives versus SLIV could provide valuable insights for evidence-based policymaking.

The cost-effectiveness of SLIV is influenced by its fixed cost structure (staff and overheads). Assuming the SLIV was not performing at capacity, attracting more children to the program could reduce the average cost per vaccination, making it more economically viable. Decision-makers should consider pairing SLIV with other cost-effective strategies to promote both vaccination uptake and program awareness. If SLIV proves to be cost-effective in boosting vaccination rates, it could be scaled up to other schools across the US. Moreover, economies of scale may be achieved if vaccines can be administered across multiple schools within a region without significantly raising costs. For example, if a team can serve several schools, then the costs of staff and equipment may be diluted through an increase in the number of vaccinated students, thus lowering average costs.

However, while scaling up offers potential benefits, it also poses risks. Cultural differences across regions may affect the acceptability of the SLIV program, which could impact its effectiveness and adoption rates.^25–27^ Furthermore, the study suggests that SLIV and practice-based vaccinations complement each other. But if scaling up makes them substitute, expanding SLIV might lead to “cannibalization”,^28^ where individuals shift from primary care vaccinations to SLIV. If the increase in SLIV uptake is not enough to reach the breakeven point, where SLIV would become cheaper than practice-based vaccinations, then this may result in higher costs per vaccinated child.

### Recommendations for future research

To complement the understanding of cost-effectiveness, it is crucial to explore why children and parents made their specific choices regarding SLIV. Qualitative studies could provide insights into why children were vaccinated at school or why their parents opted for primary care practices despite (or due to) the SLIV program.^29^

Additionally, understanding the heterogeneity in SLIV and SLIV-induced practice-based vaccination adherence is important. For instance, how does adherence vary by gender, ethnicity, income, and other socioeconomic factors?^25,27,30,31^ Would different interventions be more effective for different sub-groups? These analyses could shed light on the program’s impact on vaccination inequalities.^32–34^

It would also be important to compare the costs of vaccinating an extra child against its benefits (e.g., avoided hospital visits, lower work and school absence). Future research should draw on existing literature^24^ and/or new data to model whether the additional vaccinations through SLIV provide enough benefits to outweigh the costs. Future research efforts should also compare SLIV with other interventions aimed at increasing vaccination coverage.^35,36^

Testing the resilience of SLIV in new contexts and over time is essential to determine if its efficacy is maintained or if vaccination rates fluctuate, either increasing (e.g., through growing recognition) or decreasing (e.g., due to losing novelty).^37–39^

Evaluating SLIV in other localities is also important to understand if these effects are generalizable across the USA or specific to Monroe County, New York.

Finally, in the study by Yoo et al. (2019), web consent and paper-based consent were applied differently between primary and secondary schools. For a correct comparison of cost-effectiveness, web consent should also be tested consistently across schools, either elementary against elementary or secondary against secondary schools.

### Conclusions

This commentary critically appraised the economic evaluation by Yoo et al. (2019) on the cost-effectiveness of the SLIV program. The study was found to be methodologically robust, with only minor concerns noted in the discussion of certain results. Specifically, the study does not explore the mechanisms by which spillover effects influence cost-effectiveness and lacks a comparative discussion of SLIV against other strategies aimed at increasing vaccination uptake among school-aged children. A better understanding of public preferences for SLIV and the resulting spillover effects could enhance the effectiveness of programs aimed at boosting influenza vaccination rates among school-aged children.

## Figures and Tables

**Table 1:** Critical appraisal tool

#	Question	Answer
	**A. Rationale**	
**A1**	**Is there a clear statement of the decision problem?**	⊠ Yes □ No □ Unclear □ Not applicable
	**B. Effectiveness**	
**B1**	**Was the effectiveness of the intervention established on a systematic review?**	□ Yes ⊠ No □ Unclear □ Not applicable
	**C. Comparators**	
**C1**	**Was a comprehensive description of the competing alternatives given? (i.e. can you tell who did what to whom, where, and how often)**	⊠ Yes □ No □ Unclear □ Not applicable
	**D. Model perspective and structure**	
**D1**	**Is the perspective of the model clearly stated?**	⊠ Yes □ No □ Unclear □ Not applicable
**D2**	**Are the model structure and its assumptions appropriate and do they fit with the clinical theory of the disease process?**	⊠ Yes □ No □ Unclear □ Not applicable
	**E. Costs**	
**E1**	**Were all important and relevant costs for each alternative identified?**	⊠ Yes □ No □ Unclear □ Not applicable
**E2**	**Were costs measured and valued appropriately?**	⊠ Yes □ No □ Unclear □ Not applicable
	**F. Outcomes**	
**F1**	**Were all important and relevant outcomes for each alternative identified?**	⊠ Yes □ No □ Unclear □ Not applicable
**F2**	**Were outcomes measured and valued appropriately?**	□ Yes □ No ⊠ Unclear □ Not applicable
	**G. Analysis**	
**G1**	**Was the analysis designed appropriately?**	⊠ Yes □ No □ Unclear □ Not applicable
**G2**	**Were the methods and assumptions used to extrapolate short-term** **results to final outcomes been documented and justified?**	□ Yes □ No □ Unclear ⊠ Not applicable
**G3**	**Was uncertainty in the estimates of costs and outcomes adequately characterised?**	⊠ Yes □ No □ Unclear □ Not applicable
	**H. Presentation and discussion of findings**	
**H1**	**Were the results interpreted appropriately?**	⊠ Yes □ No □ Unclear □ Not applicable
**H2**	**Did the study discuss the generalisability of the results to other** **settings and patient/client groups?**	⊠ Yes □ No □ Unclear □ Not applicable
**H3**	**Did the study allude to, or take account of, other important factors in** **the choice or decision under consideration (e.g. distribution of costs** **and consequences, relevant ethical issues, or issues of implementation)?**	⊠ Yes □ No □ Unclear □ Not applicable
	**I. Transferability to UK NHS**	
**I1**	**Are the health care system, setting, comparator and patient group** **comparable to the UK and to the NHS?**	⊠ Yes □ No □ Unclear □ Not applicable
